# Gastrointestinal Residue Removal Using a Balloon Overtube under Ultrathin Endoscopic Navigation: Ex Vivo and In Vivo Experimental Studies

**DOI:** 10.3390/jcm10173796

**Published:** 2021-08-25

**Authors:** Kazuhiro Kozuka, Hideki Kobara, Noriko Nishiyama, Shintaro Fujihara, Naoya Tada, Takanori Matsui, Tadayuki Takata, Taiga Chiyo, Nobuya Kobayashi, Tingting Shi, Koji Fujita, Joji Tani, Tatsuo Yachida, Akihiro Kondo, Kensuke Kumamoto, Keiichi Okano, Akira Nishiyama, Kazushi Deguchi, Tsutomu Masaki

**Affiliations:** 1 Department of Gastroenterology and Neurology, Faculty of Medicine, Kagawa University, Kagawa 761-0793, Japan; kobara@med.kagawa-u.ac.jp (H.K.); n-nori@med.kagawa-u.ac.jp (N.N.); joshin@med.kagawa-u.ac.jp (S.F.); n-tada@med.kagawa-u.ac.jp (N.T.); tk-matsui@med.kagawa-u.ac.jp (T.M.); t-takata@med.kagawa-u.ac.jp (T.T.); t_chiyo@med.kagawa-u.ac.jp (T.C.); nobuyak@med.kagawa-u.ac.jp (N.K.); shitingtingc@med.kagawa-u.ac.jp (T.S.); 92m7v9@med.kagawa-u.ac.jp (K.F.); georget@med.kagawa-u.ac.jp (J.T.); tyachida@med.kagawa-u.ac.jp (T.Y.); kdeguchi@med.kagawa-u.ac.jp (K.D.); tmasaki@med.kagawa-u.ac.jp (T.M.); 2 Department of Gastroenterological Surgery, Faculty of Medicine, Kagawa University, Kagawa 761-0793, Japan; kondo.akihiro.z4@kagawa-u.ac.jp (A.K.); kumamotk@med.kagawa-u.ac.jp (K.K.); kokano@med.kagawa-u.ac.jp (K.O.); 3 Department of Pharmacology, Faculty of Medicine, Kagawa University, Kagawa 761-0793, Japan; nishiyama.akira@kagawa-u.ac.jp

**Keywords:** gastrointestinal residue, endoscopic removal, gastrointestinal bleeding, balloon overtube

## Abstract

Pooled gastric residues involving blood clots and food interrupt appropriate endoscopic intervention, leading to poor outcomes in endoscopic hemostasis and lifesaving. However, procedures and devices that enable the effective removal of gastrointestinal residues remain unsatisfactory. This study aimed to evaluate the efficacy and safety of our developed suction method in ex vivo and in vivo studies. We created a hand-made device with a large suction diameter, consisting of a balloon overtube and an ultrathin endoscope for navigation. In the ex vivo study, we compared the success rate and the suctioning time for four types of simulated residue between a standard endoscope and our device. Our device had s significantly higher suction ability and a shorter procedure time than the standard endoscope. The subsequent in vivo animal study involved five beagle dogs that were administered with food jelly to mimic gastric residue. Suction was performed twice for five dogs (ten attempts). The outcome measure was the successful procedure rate; secondary outcomes were procedure-associated complications and procedure time. The procedure was successful in all attempts, without any complications. The mean procedure time was 5 min. This novel method enabled the efficient and safe removal of gastric residue, and our findings will likely lead to the development of the instrument.

## 1. Introduction

Despite the remarkable developments in upper gastrointestinal endoscopy-related procedures in recent years, more advanced and delicate endoscopic techniques are still required. Clinicians often encounter situations in which endoscopic treatment is made difficult by the presence of gastrointestinal (GI) residues such as blood clots and food [1,2,3]. The accumulation of a large amount of GI residue interferes with the endoscopic operation field and may lead to a decreased therapeutic completion rate and increased rate of complications. Therefore, it is necessary to remove the GI residue and secure a sufficient operation field for safe treatment. In addition, GI residue can make it difficult to identify the bleeding site, especially in cases which involve a lot of bleeding. If the hemostatic procedure is prolonged, the mortality rate may increase.

The fundus and upper-body are common sites of hemorrhagic gastric ulcers, with 22.5% of all gastric ulcers occurring at these sites [4]. Blood clots and food residues are also likely to accumulate in these sites, owing to anatomical features and gravity. In clinical practice, these GI residues cannot be effectively removed using a standard endoscope with a 2.8–3.2-mm diameter forceps channel. Therefore, there is a need for a method that enables the rapid removal of a large amount of GI residue. At present, such techniques for GI residue removal remain clinically undeveloped. We previously developed a novel GI residue removal method using an existing double-balloon overtube under the guidance of ultrathin endoscopy and reported its effectiveness in preliminary animal experiments [5]. The present study aimed to validate the feasibility, safety, and effectiveness of our novel GI residue removal method.

## 2. Materials and Methods

### 2.1. Experimental Equipment

**Figure 1:**
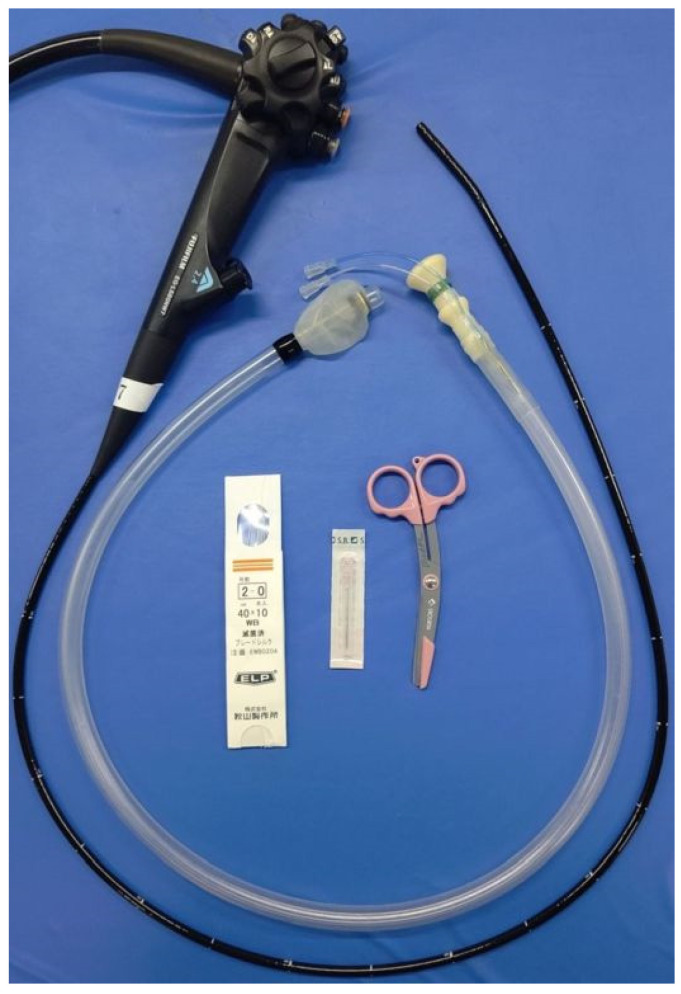
Experimental equipment.

The experiment was performed using an ultrathin endoscope (EG-L580NM7, FUJIFILM, Tokyo, Japan), a CO2 insufflation device (GW-100, FUJIFILM, Tokyo, Japan), a double-balloon overtube (TS-12140, FUJIFILM, Tokyo, Japan), single-use biopsy forceps (Radial Jaw 4 Gastropediatric Biopsy Forceps, Boston Scientific, Marlborough, MA, USA), silk thread, scissors, 18-G needle, and vinyl tape (Figure 1).

### 2.2. Preparations

For navigation, an ultrathin endoscope (caliber, 5.9 mm) was inserted into the double-balloon overtube lumen (caliber, 10.8 mm), resulting in a hand-made suction device with a large suction diameter.

**Figure 2:**
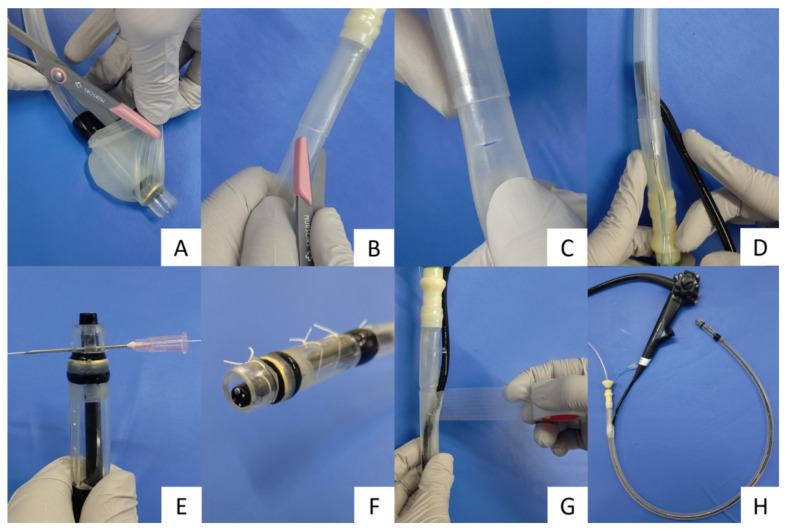
Procedure for creating a gastric residue suction device. (**A**) Remove the overtube-mounted balloon section using scissors. (**B**,**C**) Make a 10 × 10 mm cross incision with scissors 10 cm from the base of the overtube. (**D**) Insert the ultrathin endoscope into the tube through the cross incision. (**E**,**F**) Fix the scope to the tube with silk thread 2 mm from the distal tip. (**G**) Reinforce the cross-incision with vinyl tape. (**H**) Connect the suction tube to the base of the overtube.

Step 1: Removal of the overtube-mounted balloon part using scissors (Figure 2A);
Step 2: A 10 × 10 mm cross incision was made with scissors 10 cm from the base of the overtube, and the ultrathin endoscope was inserted into the tube. For endoscopy, sterile lubricating gel was applied to the opening so that the endoscope could be easily inserted (Figure 2B–D);
Step 3: The endoscope was fixed 2 mm from the distal tip of the overtube through the incision hole and was connected by using silk thread at four points. In detail, the tube was pierced with an 18-G needle to avoid damaging the scope and was fixed by passing a silk thread through the needle (Figure 2E,F);
Step 4: The cross-incision was reinforced with vinyl tape to prevent the loss of suction pressure (Figure 2G);
Step 5: The suction tube was connected to the base of the overtube (Figure 2H).

### 2.3. Ex Vivo Study: Comparison of Suction Ability between a Standard Endoscope and Our Hand-Made Device

**Figure 3:**
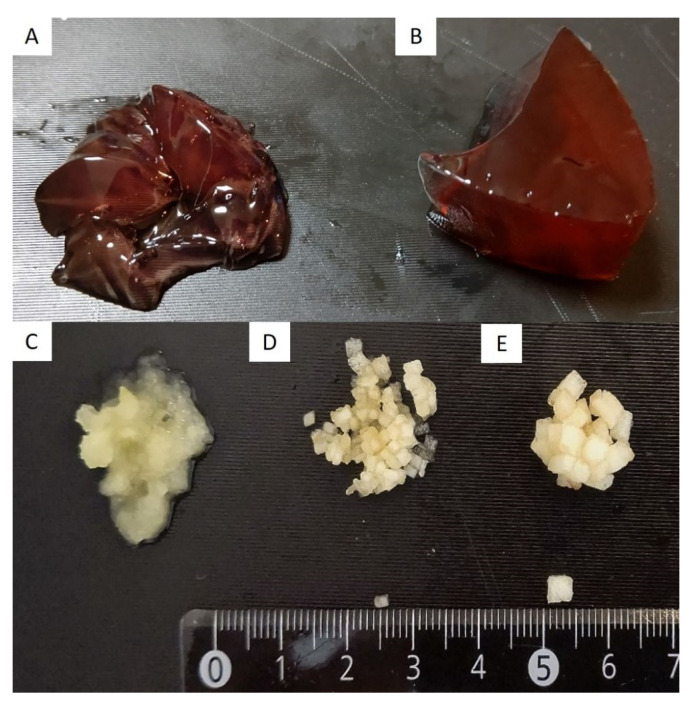
Four types of simulated residues to be suctioned. (**A**) Soft jelly made of agar. (**B**) Hard jelly made of konnyaku. (**C**) Grated apple. (**D**) 1 mm^2^ apple pieces. (**E**) 3 mm^2^ apple pieces.

**Figure 4:**
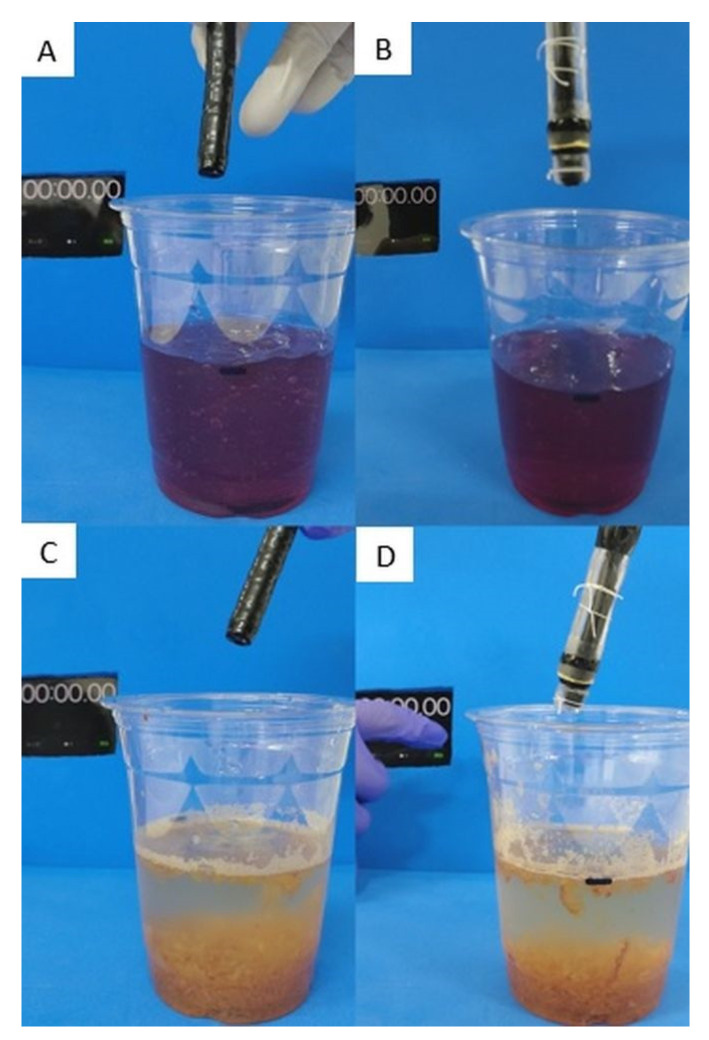
Suction test for a soft jelly made of agar: (**A**) standard endoscope vs. (**B**) our hand-made device. Suction test for grated apple: (**C**) standard endoscope vs. (**D**) our device.

In the ex vivo study, a standard endoscope with a 3.2 mm diameter forceps channel (GIFQ260J; Olympus Co., Tokyo, Japan), which was most applicable in clinical practice, was used for comparison with our device. As simulated residues, we prepared four types of food, namely a soft jelly made of agar, hard jelly made of konnyaku that is a taro-like potato, grated apples, 1 mm^2^ apple pieces, and 3 mm^2^ apple pieces (Figure 3). 200 mL of soft or hard jelly was injected into a transparent cup, and 20 g of grated or 1 or 3 mm^2^ apple pieces were placed on the bottom of a transparent cup. Water was then added until the volume was similar to that of the jelly groups (200 mL). We compared the success rate and the procedure time for suctioning the four types of residue by using a standard endoscope and our device (Figure 4). Each object was suctioned three times each using the standard endoscope or our device. When the scope or tube was obstructed during an attempt, the channel was cleared with an air flush.

### 2.4. In Vivo Animal Study

Based on the efficacy of our developed device through the ex vivo study, this animal experimental study was conducted in a single institution from December 2020 to March 2021. Five 11–13-month-old female beagle dogs (Hokuzan Rabesu Co., Nagano, Japan) were included in the present study. These dogs were administered with 150 mL of food jelly to create a model of gastric residue. The food jelly was administered until the greater curvature in the upper stomach became invisible. We confirmed that a standard endoscope could not suction this jelly. Our newly developed removal method was performed twice for each dog, with a 10-min interval between removal attempts. A total of 10 residue removal procedures were performed in the five dogs. The suction pressure was set at 40 kPa.

The dogs were fasted for 24 h before the intervention. Procedures were performed under general anesthesia with intubation. Three hours after the procedure, the dogs received water and returned to a normal diet. They were kept in a pathogen-free facility under controlled temperature (24 ± 2 °C) and humidity (55% ± 5%), with a 12-h light/dark cycle. This animal experiment conformed to the Regulations and Guidelines for Animal Experiments and was approved by the Institutional Review Board of Kagawa University (registration no. A337, approval no. 21640).

**Figure 5:**
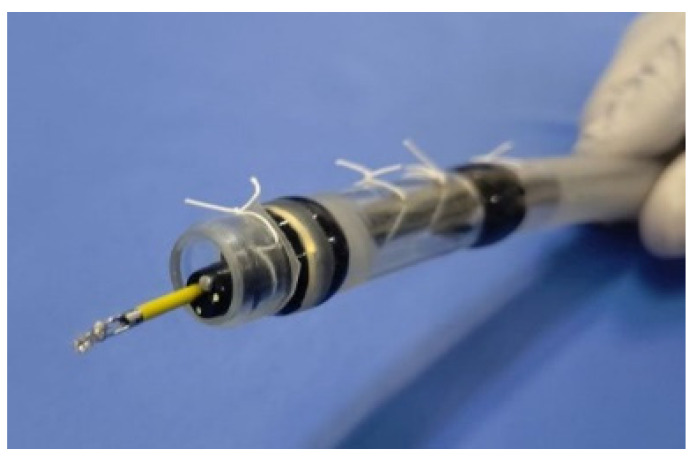
The biopsy forceps inserted via the forceps channel of the endoscope.

**Figure 6:**
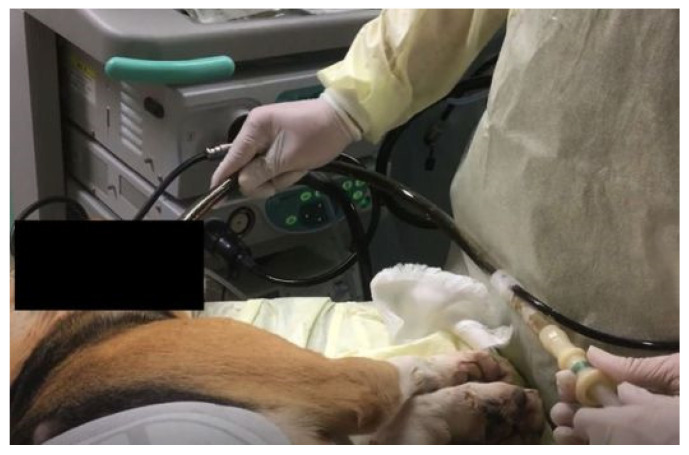
Outside condition during suction.

**Figure 7:**
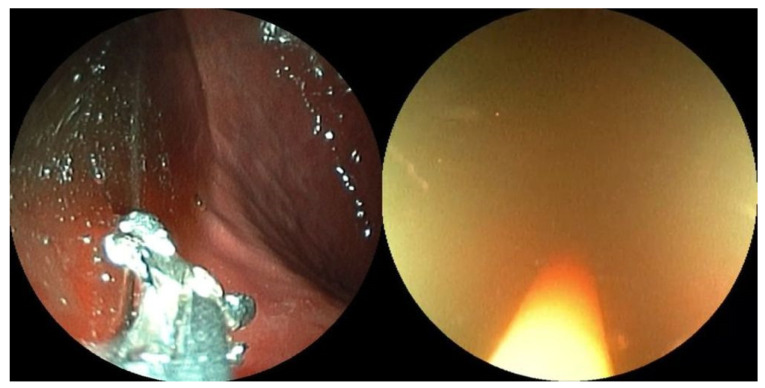
A continuous clear view is achieved by using biopsy forceps to push the mucosa.

**Figure 8:**
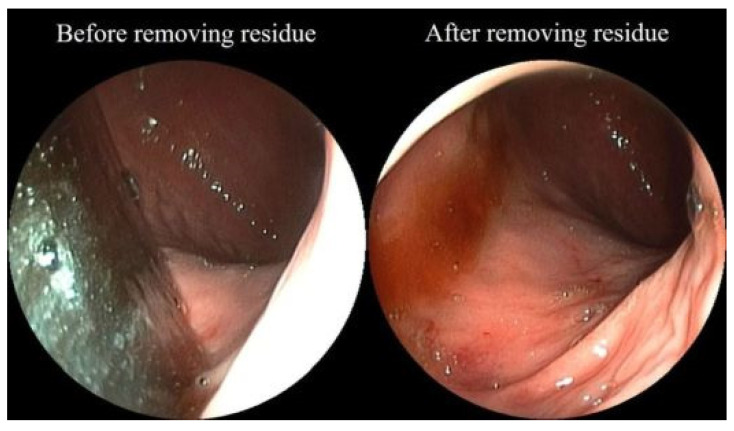
Endoscopic images showing the operation field before (left) and after (right) the application of the suction method.

Experimental Procedure (Video S1):

Step 1: The manufactured overtube was inserted into the stomach under the guidance of an ultrathin endoscope;
Step 2: After the visualization of the simulated GI residue under ultrathin endoscopy, the biopsy forceps were inserted via the forceps channel of the endoscope (Figure 5). The biopsy forceps were pressed on the superficial mucosa to prevent suctioning the mucosa;
Step 3: The GI residue was suctioned under 40 kPa negative pressure while the targeted residue was visualized using the ultrathin endoscope (Figure 6);
Step 4: An appropriate distance between the tube and superficial mucosa was maintained using the biopsy forceps (as described in Step 2), leading to continuous and effective suction (Figure 7);
Step 5: Successful suction was completed when the mucosal membrane hidden by the GI residue appeared on the greater curvature side (Figure 8).

### 2.5. Outcome Measures in the Ex Vivo Study

The primary outcome was a comparison of the successful removal rate for the four types of simulated residues between using a standard endoscope and our hand-made device. Removal was defined as successful when the entire bottom of the cup was visible after suctioning. Removal was defined as a failure when the suction did not proceed at all for more than 5 min. The secondary outcome was a comparison of procedure time between the two devices, which was defined as the duration from the start of suction to clear visualization of the entire bottom of the cup.

### 2.6. Outcome Measures in the In Vivo Animal Study

The primary outcome was the successful GI residue removal rate. Removal was defined as successful when the upper greater curvature folds became visible after suctioning. In our previous experiment, we confirmed that the folds became invisible when the jelly measured any higher than 150 mL. Therefore, we defined the appropriate amount of jelly as 150 mL. The secondary outcomes were procedure-associated complications involving mucosal damage and procedure time.

### 2.7. Statistical Analyses

All statistical analyses were performed using GraphPad Prism 7.0 (GraphPad Software, Inc., LA Jolla, CA, USA). The procedure time was described as mean ± standard deviation (SD). Comparisons of two groups between the standard endoscope and our device were performed by two-way ANOVA. *p* < 0.05 was considered significant.

## 3. Results

### 3.1. Results of the Ex Vivo Study

With the soft jelly and grated apple, the successful removal rate for both the standard endoscope and our hand-made device was 100% in each of the three attempts. With the hard jelly and 1 mm^2^ apple pieces, the rates were 0% for the standard endoscope and 100% for our device in each of the three attempts with each device. With the 3 mm^2^ apple pieces, the rate was 0% for both devices for all three attempts (Table 1).

**Figure 9:**
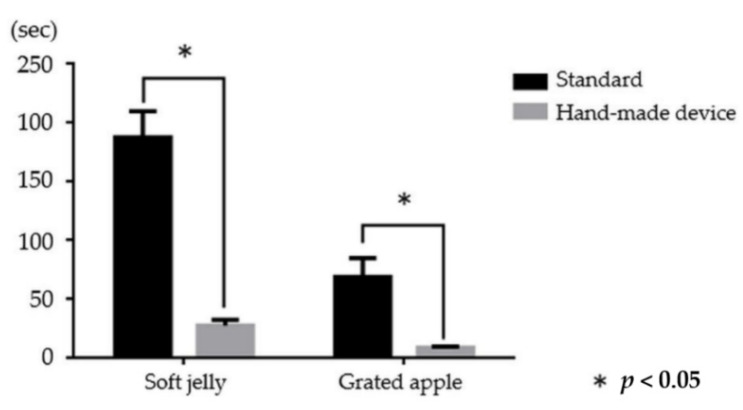
Comparison of the procedure time between a standard endoscope and our hand-made device for suctioning soft jelly and grated apple. *: *p* < 0.05.

We compared the procedure time for successfully suctioning soft jelly and grated apple using both devices (Table 2). With the soft jelly, the procedure time was significantly shorter using our device versus the standard endoscope (mean ± standard deviation (SD): 28 ± 5 vs. 188 ± 22 s, respectively; *p* < 0.05). With the grated apple, the procedure time was also significantly shorter using our device versus the standard endoscope (mean ± SD: 9 ± 1 vs. 69 ± 16 s, respectively; *p* < 0.05) (Figure 9).

### 3.2. Results in Experimental Animal Study

The successful removal rate was 100% for each of the 10 attempts. There were no complications, including bleeding, perforation, and mucosal damage. The mean procedure time was 303 ± 137 s (Table 3).

## 4. Discussion

This ex vivo and in vivo animal study was performed to verify the efficacy and safety of our newly developed endoscopic method for removing a large amount of gastric residue to maintain the operation field. The ex vivo study that designed four types of simulated residue, comprising gels or solid substances, was the first and novel study evaluating the usefulness of an endoscopic food residue removal method. There were four main findings. Firstly, our method was superior to suctioning using a standard endoscope in terms of successful removal and procedure time in ex vivo study; however, our method provided a limited ability when suctioning a 3 mm^2^ solid substance. Secondly, the large-diameter suction channel using the overtube was also efficacious for GI residue removal in the in vivo animal study. Thirdly, the ultrathin endoscope played an important role by enabling the aspiration of the residue under direct vision. Fourthly, the biopsy forceps worked well in achieving continuous suction.

The conditions under which blood clots accumulate in the GI tract are peptic ulcer bleeding during emergency endoscopy and massive bleeding during endoscopic submucosal dissection for early GI cancer. These conditions are often encountered in clinical practice. In addition, the accumulation of food residue occurs due to dyspepsia and GI dyskinesia, which makes it difficult to visualize the GI lesions and forces the postponement of scheduled endoscopic surgery. Poor visualization of the GI lumen is a major factor resulting in the discontinuation of endoscopic treatment. To overcome this issue, the patient is conventionally changed from the left to the right lateral decubitus position. This results in residual movement due to gravity and secures the operation field. However, the maneuverability in the right lateral decubitus position is poorer than that in the left position, conventionally used by the right-handed operator. In addition, the right repositioning has a minimal effect when there is a large amount of residue. Therefore, it is desirable to develop a device capable of removing pooled GI residues quickly and efficiently. We developed a hand-made device using an overtube and an ultrathin endoscope as described in the present study.

According to the Japanese national database, the number of patients with gastric ulcers in 2017 was approximately 16 per 100,000 population [6]; this number is a quarter of that reported in 1984. The main causes of gastric ulcers are *Helicobacter pylori* infection and the administration of non-steroidal anti-inflammatory drugs/antiplatelet drugs (mainly low-dose aspirin). The number of patients infected with *H. pylori* is decreasing due to the eradication and the widespread use of proton-pump inhibitors [4]. However, the number of older adults in Japan is rapidly increasing, and the risk of drug-induced ulcers is relatively high because many older adults take non-steroidal anti-inflammatory drugs, anticoagulants, and antithrombotic drugs. Therefore, endoscopic hemostasis for upper GI ulcer bleeding is often required [6].

Recent endoscopic advancements have provided excellent outcomes in the management of GI ulcer bleeding [7]. Endoscopic clipping and drug injection have been conventionally applied since endoscopy was first introduced [8,9]. In recent years, the coagulation hemostatic method using hemostat forceps has become popular [10]. Moreover, polyglycolic acid sheets and over-the-scope clips have been introduced as new hemostatic methods [11,12]. While endoscopic hemostasis is a well-established procedure, it requires a secured operation field. However, there are few previous reports on the method used for the endoscopic removal of gastric residues.

It is difficult to blindly remove gastric residue effectively using a suction tube without the use of an endoscope, as the suction tube often accidentally aspirates the mucosal membrane. There are several reports of residue removal methods without endoscopic intervention. One method involves the removal of residues by gastric lavage, but the success rate of this procedure is only 11.8% [13]. Another method is to make the patient ingest a large amount of water in a short time; however, the success rate of this method is only 30%, and the procedure may cause patient discomfort and may not be suitable for emergency endoscopy [14]. There are also reports of methods that promote gastric residue excretion by using drugs such as somatostatin and erythromycin [15,16]. However, these drug effects take a relatively long time to take effect, and the removal success rate is inadequate (64%). An endoscopic residue removal method has been reported for the esophagus [17]. In this method, a gastric tube is fixed to the side of the endoscope, and suction is performed. However, the stomach has a larger amount of residue than the esophagus. Furthermore, if we simply enlarge the suction port, the mucosal membrane can become attached to it, making it difficult to suction the residue. Therefore, GI residue removal requires endoscopic navigation under direct vision.

**Figure 10:**
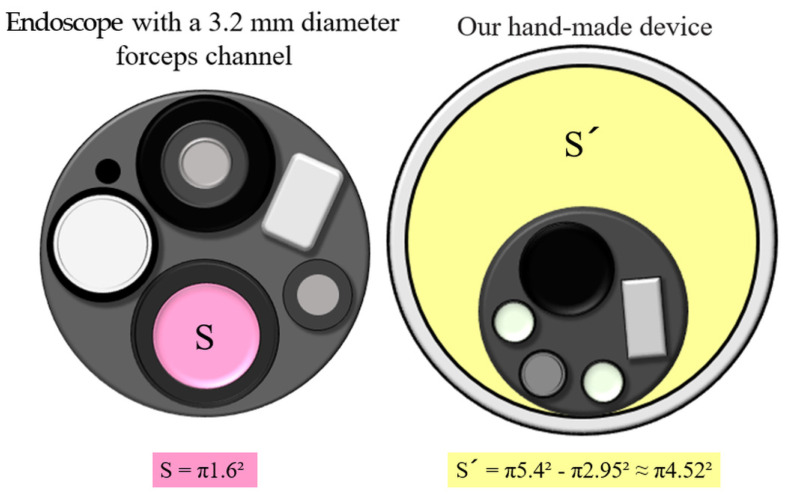
Comparison of suction area (S) with the standard endoscope and (S’) our hand-made device.

The residue suction method used in this study satisfied this requirement. Installing the ultrathin endoscope in the overtube enabled us to effectively perform endoscopic suctioning. In addition, the diameter of the overtube was 10.8 mm. Even if an ultrathin endoscope with a 5.9 mm diameter is used, a large suction diameter of 4.9 mm is secured in our method. In contrast, the maximum suction channel diameter among clinically available standard endoscopes is 3.2 mm. When calculating the suction area between two devices, a more significant difference appeared, as illustrated in Figure 10. The suction areas were as follows: standard endoscope vs. our device: π 1.6 mm^2^ vs. π 4.52 mm^2^. Thus, our device, with a suction tube and an inner diameter of 9-mm, is more suitable for suctioning GI residues. The results of the ex vivo study support a difference in suction areas between the two devices. Our hand-made device had a significantly higher suction capacity and required a shorter procedure time than with the standard endoscope. Regarding successful residue removal, while the standard endoscope had limited suctioning for three types of residue (hard jelly, and 1 and 3 mm^2^ apple pieces), our method showed limited ability only for 3 mm^2^ apple pieces. Larger solid substances, such as the 3 mm^2^ apple pieces caused intraluminal obstruction, although these apple pieces could be aspirated into the lumen of the suction channel. Further innovations and dedicated devices are required to resolve this issue.

Another advantage of our method is that a constant distance between the mucosal membrane and the overtube can be maintained by inserting biopsy forceps through the endoscope; this decreases the likelihood of suction error and enables continuous suction under clear visualization. Our removal method was successful, experiencing no complications and entailing an acceptable mean procedure time (5 min) in all 10 attempts in the 5 dogs. However, the biopsy forceps applied for achieving continuous suction has a few potential risks of complication such as mucosal injury, though no obvious complications appeared in the present study. The maneuver, pushing biopsy forceps on the mucosa, is similar to checking the cushion sign of subepithelial lesions using the forceps. Thus, the maneuver seems less invasive. Without the forceps, the overtube may frequently induce larger mucosal damage because of the suction error. Consequently, the combination of biopsy forceps and ultrathin endoscope is considered an effective tool for promptly suctioning the residue and preventing the mucosal injury by suction errors under direct vision.

The double-balloon overtube is clinically used for enteroscopic delivery. The overtube may potentially cause mucosal injury as it passes through the narrow anatomical areas of the pharyngo-esophageal or esophago-gastric junction. However, in the present animal study, there were no complications such as bleeding, perforation, and mucosal damage. This indicates the safety of our gastric residue removal method.

To apply this suction method in clinical practice, several issues should be discussed.

Although the old type of ultrathin endoscope, which could be used for the animal experiment, had relatively poor image quality, newly developed ultrathin endoscopes with high image quality are clinically available. Thus, the image quality of ultrathin endoscope would be not clinically concerned. If the treatment equipment is not used immediately, the procedure time for hemostasis may be prolonged. Therefore, it is more desirable to perform suction and hemostasis simultaneously. A novel hemostatic forceps with a 2.3 mm diameter (RAICHO, KANEKA, Osaka, Japan), suitable for the ultrathin endoscopes (FUJIFILM, Tokyo, Japan) with a 2.4 mm diameter forceps scope channel, has been made recently available in clinical practice. Several authors reported that the RAICHO was efficacious for endoscopic hemostasis during endoscopic submucosal dissection using the ultrathin endoscope [18,19]. Accordingly, the simultaneous procedure seems possible. A further study is needed to verify the efficacy of this method. When the patient presents a great deal of bleeding in unstable vital status, the use of the suction device should be recommended only under general anesthesia in order to maintain the safety of the patient. Meanwhile, the suction device is originated in the overtube used clinically for enteroscopy, and the tube diameter is same. Thus, the device-associated complications are expected to be low, similarly to that of enteroscopy use.

The present study had the following limitations: the procedures were performed by a single operator; this was an in vivo animal study of a self-created residue model; the effectiveness of this procedure in removing solid food residues was not verified. There was another limitation. While the main purpose of this study was to maintain the operation field by removing blood and foods using our developed device, the clinical effect of whether a clear operation field contributes to the success rate and procedure time of endoscopic hemostasis remains insufficient. In contrast, an unclear operation field is caused by poor preparation and intraoperative bleeding during endoscopic tumor resection. These situations force us to postpone the operation and interrupt the consecutive procedure. Thus, measures to quickly remove the residue and ensure a clear endoscopic field are necessary and meaningful in clinical practice. A clinical study is now scheduled to evidence this argument. Furthermore, a comparable model of blood clots related to medical procedures was absent in this study. Prior to the start of the study, we considered the introduction of human red blood cell (RBC) products for blood transfusion. In the ex vivo model, because the RBC easily harden in the room air, we could not individually create the same model of blood clots. In the animal model, inserting human blood into the animal raised ethical issues. Consequently, we applied gelatinous jelly as simulated blood clots.

In conclusion, this study demonstrated that our novel GI residue removal method may be effective for the removal of a large amount of GI residue. The present findings may lead to the development of an instrument based on the concept of this hand-made device.

## Figures and Tables

**Table 1:** Successful residue removal rate in the ex vivo study (three attempts for each residue).

Endoscope	Soft Jelly	Hard Jelly	Grated Apple	1 mm^2^ Apple Pieces	3 mm^2^ Apple Pieces
Standard	100	0	100	0	0
Hand-made device	100	100	100	100	0

Values are the success rate (%).

**Table 2:** Procedure time in the ex vivo study.

Endoscope	Soft Jelly	Hard Jelly	Grated Apple	1 mm^2^ Apple Sections	3 mm^2^ Apple Sections
Standard	188 ± 22	-	69 ± 16	-	-
Hand-made device	28 ± 5	377 ± 34	9 ± 1	8 ± 2	-

Values are the procedure time (sec), mean ± standard deviation (SD).

**Table 3:** Results of the endoscopic gastric residue removal method in a total of 10 attempts in 5 beagle dogs.

Case	Attempt	Residue Volume (mL)	Suction Pressure (kPa)	Procedure Time (sec)	Successful Residue Removal	Complications
1	1	150	40	235	Yes	None
	2	150	40	265	Yes	None
2	3	150	40	540	Yes	None
	4	150	40	200	Yes	None
3	5	150	40	390	Yes	None
	6	150	40	130	Yes	None
4	7	150	40	210	Yes	None
	8	150	40	352	Yes	None
5	9	150	40	530	Yes	None
	10	150	40	180	Yes	None

## Data Availability

Data are not publicly available due to protection of personal data and medical confidentiality.

## Supplementary Materials

The following are available online at https://www.mdpi.com/article/10.3390/jcm10173796/s1, Video S1: In Vivo Experiments with Beagle Dogs.
